# Probiotic yogurt Affects Pro- and Anti-inflammatory Factors in Patients with Inflammatory Bowel Disease 

**Published:** 2013

**Authors:** Mahdi Shadnoush, Rahebeh Shaker Hosseini, Yadollah Mehrabi, Ali Delpisheh, Elham Alipoor, Zeinab Faghfoori, Nakisa Mohammadpour, Jalal Zaringhalam Moghadam

**Affiliations:** a *National Nutrition and Food Technology Research Institute, Faculty of Nutrition Sciences and Food Technology, Shahid Beheshti University of Medical Sciences, Tehran, Iran.*; b *Department of Epidemiology, School of Public Health, Shahid Beheshti University of Medical Sciences, Tehran, Iran. *; c *Department of Clinical Epidemiology, Ilam University of Medical Sciences, Ilam, Iran.*; d *School of Nutritional Sciences and Dietetics, Tehran University of Medical Sciences, Tehran, Iran. *; e *Students Research Committee, Faculty of Nutrition, Tabriz University of Medical Science, Tabriz, Iran. *; f *Department of Physiology, Shahid Beheshti University of Medical Sciences, Tehran, Iran. *

**Keywords:** Inflammatory bowel disease, Probiotic, Yogurt, Cytokine

## Abstract

Inflammatory bowel disease (IBD) is an irregular response of immune system accompanied with different inflammatory manifestations including alterations in cytokines. Probiotics are non-pathogenic organisms with probable effects in various conditions such as inflammation. The present study hypothesized whether oral intake of *bifidobacterium *and *lactobacillus *in form of probiotic yogurt may represent an immunomodulatory effect in IBD patients.

Overally, 210 patients in remission phase and 95 healthy people were recruited. Patients were randomly divided into two groups of either 250 grams of probiotic yogurt (PI) or 250 grams of plain yogurt (PC) daily for 8 weeks. The healthy control group (HG) also received probiotic yogurt as noted. The serum levels of cytokines TNF-*α*, IL-6, IL-1*β*, IL-10 and CRP levels were measured at baseline and at termination time.

A significant difference was observed between intervention groups of PI and PC with HG group (p < 0.05). After the intervention, serum levels of IL-1*β*, TNF-*α *and CRP were significantly decreased in PI group compared to their baseline values and intervention groups. The serum levels of IL-6 and IL-10 increased significantly after the intervention compared to baseline values and PC levels (all p-values < 0.05).

Intestinal homeostasis is a balance between pro and anti-inflammatory responses of intestinal immunocytes and could be maintained by probiotics.

## Introduction

Inflammatory bowel disease(IBD) is comprised of two enteritis disorders (ulcerative colitis(UC) and crohn’s disease (CD) with poorly understood etiologies that have similar disease characteristics but have different advent (1). 

IBD was previously considered as a health threatening problem in western industrialized countries, particularly in northern areas of America and Europe with a rapid spread during the last decades worldwide (2, 3). In Iran, no real prevalence for IBD has been specified, but the existed data shows a growing trend in CD in particular (3, 4).

Both genetic and environmental factors are considered as potential risk factors for IBD, with no consensus about the priority of these factors. A pattern similar to diabetes has been suggested, which means that probably people who have genetic background and environmental exposures exacerbate disease progression (1, 5). According to a Japanese study, environmental factors are more imperative, as genetic background will not change notably whereas disease incidences are increased significantly (2). Chronic inflammatory disease is an irregular response of immune system (6). The inability of attenuating inflammation in gut allows the irritating agents to stimulate immune system and cause inflammatory reactions that can result in further pathophysiological disorders. Accordingly, increased immune cells and enhanced production of pro-inflammatory Cytokines (CKs) including interleukin (IL)-1, IL-6, IL-8, IL-12 and tumor necrosis-*α *(TNF-*α*) have been reported (6-9). Based on functions, Cytokines are classified into two categories; proinflammatory and anti-inflammatory CKs. CKs carry signals between immune, epithelial and mesenchymal cells (10, 11). Assessment of inflamed gastrointestinal mucosa in IBD patients has shown an enhanced expression of these CKs which is resulting to diarrhea or constipation (11-13). Nowadays, medications that are using as therapeutic agents in IBD patients like corticosteroids, biological treatments such as anti-cytokine drugs, immune affecting drugs and others are effective in disease maintenance partially, and there is no definite treatment approach for IBD because of its diverse phenotypes (14, 15). 

Probiotics are alive non-pathogenic organisms that have been identified since last century for their probable benefits (16), such as inflammatory and non-inflammatory conditions, allergies, arthritis, ectopic eczema, infections, the gastrointestinal epithelium health and *etc. *(16, 17). Meanwhile, attenuating lactose intolerance, improvement in lipid profile and cholesterol levels, improvement in nutritional values of food items and quality of digestion have also been considered (18). Both experimental and clinical studies have examined different strains of probiotic bacteria and reported different results about their efficacy (19). The *Bifidobacterium BB-12 *and *Lactobacillus acidophilus La-5 *as probiotics strains might have immunomodulatory effects (20).

A definite etiology has not been set for IBD yet and the recognition of efficient treatment approaches is difficult (21). Due to increased incidence in Iran (4), serious complications and future disease burden are expected. The present study hypothesized whether oral intake of probiotics such as *Bifidobacterium *and *Lactobacillus *in form of probiotic yogurt may represent an immunomodulatory effect in IBD patients. 

## Experimental

The participants in the study were selected from IBD patients in remission who were referred to either gastrointestinal hospital wards or clinical research centers. The diagnosis was confirmed by using a gastroenterologist. Healthy control group was chosen among people who met inclusion criteria without IBD history. An informed consent was considered as a basic entrance criterion. The present trial was approved by the Ethical Committee of Shahid Beheshti School of Medical Sciences, Tehran-Iran. The present randomized double-blind clinical trial was registered at the www.irct.ir, identifier: IRCT201105106431N1.

Body mass index (BMI) was calculated for all participants. Exclusion criteria was the consumption of supplements including omega-3, antioxidant vitamins (vitamin A, E, C) and the probiotic and prebiotics products 3 months preceding the study. Patients who received antibiotics, those with inflammatory diseases such as rheumatoid arthritis, infectious diseases and other gastrointestinal (GI) deficits, as well as nursing mothers and pregnant women were also excluded. 

The intervention was stopped if participants drug regiments (dose or type) were changed. Participants who didn’t consume 30% or more (≥30%) of their daily yogurt in each 10 days period were not allowed to continue the rest of intervention. 

All participants were advised to keep the previous life style including diet, exercise and smoking constant during the intervention period.

In general, 3 study groups including one healthy group and two patient groups were prepared. Overall, 210 patients were divided randomly into two groups, one received 250 grams of probiotic yogurt and the other received 250 grams of plain yogurt daily for 8 weeks. The healthy control group (n = 95) received 250 grams probiotic yogurt for the same duration.

The probiotic yogurt contained two strains; *Bifidobacterium *and *Lactobacillus *.The mean concentration was 106 colony forming units (CFU) per each gram of yogurt. All yogurts had 1.5% fat with a 20-day shelf time. The packaging of yogurts was identical for all groups and they were coded by factory in line with double blinding design. General information was gathered by a questionnaire. A three-day dietary recall for assessing food intake was assessed by a nutritionist in baseline at the end of intervention. Weights and heights were measured and 5ml blood sample were obtained after 10 - 12 h of fasting to determine serum levels of cytokines such as TNF-*α*, IL-6, IL-1*β*, IL-10 and C reactive protein (CRP) twice, at baseline and at the end of intervention in order to follow the alterations. Blood samples were freezed and serum extraction were done according to instructions; for clotting, blood samples were left for 30 minutes in serum tubes, and then centrifuged for 10 minutes at 1000 × *g*. In the next step the serum was removed and the samples stored at ≤ -20°C until the study terminated and cytokine levels were measured. IL-1*β*, IL-6, IL-10 levels were measured by Bioscience ELISA kits ( BD bioscience, Franklin Lakes, United States ) and the CRP and TNF-*α *levels were measured by Phoenix ELISA kits (Phoenix pharmaceuticals, CA, United States).

Nutrients intake was analyzed by nutritionist IV software. Mean values before and after intervention were compared by paired t-test in all groups and for data which did not follow a normal distribution Wilcoxon-signed ranks was used. Comparing the baseline mean values among groups was done by one-way ANOVA test. P-value < 0.05 was considered statistically significant.

## Results

Overally, 210 patients and 95 healthy controls were recruited. During the study period, 19 patients in PI (patients in intervention group), 15 in PC (patients in control group) and 11 in HG (healthy group) refused to continue the study or were set aside because of exclusion criteria. Consequently, the study was conducted by 86 patients in PI, 90 in PC and 84 in HG. 

The mean age of participants was 37.69 years (ranges 26 to 59 years). The statistical analysis didn’t show any significant difference among them for these values and also about sex, family history and smoking status. Mean BMI was 24.17±2.65 kg/m^2^. 

Mean energy and nutrients intakes were assessed via three recalls in initiation and at the end. According to statistical analysis, we observed that mean energy, carbohydrate, protein, total fat, cholesterol, SFA, MUFA, PUFA, vitamin D and calcium intake didn’t have significant differences between groups at baseline and at the end and also in each group before and after the intervention duration (p > 0.05). Fiber intake was significantly different between PI and PC (p < 0.05) after the intervention but when this difference was adjusted according to energy intake, it was not significant anymore (p > 0.05).

Serum levels of CRP, IL-1*β*, TNF-*α*, IL-6 and IL-10 in three study groups before and after the intervention have been demonstrated in Table 1. The differences between serum levels of cytokines and CRP in PI and PC at baseline were not statistically significant (p < 0.05), but a significant difference was observed in comparison to HG (Table 1). 

**Table 1:** Serum levels of CRP, IL-1*β*, TNF-*α*, IL-6 and IL-10 before and after the intervention

**Pro- & anti-inflammatory factors (mean ± SD)**	**PI (n=86) **	**Groups**	**HG (n=84)**
		**PC (n=90) **	
**CRP**(mg/mL)			
At baseline	8.4±3.9	7.9±3.3	1.7±1.6 a
After intervention	6.2±3.1	8.1±3.7 a	1.9±1.7 a
P values	p < 0.01	p < 0.05	P<0.05
**IL-1** ***β***(pg/mL)			
At baseline	75±4.3	72± 3.8	ND
After intervention	51±2.6	75±2.6 b	ND
P values	p < 0.01	p < 0.05	----
**TNF-** ***α***(pg/mL)			
At baseline	315±9.2	322±9.3	ND
After intervention	225±4.7	318±4.9 a	ND
P values	p < 0.001	p < 0.05	----
**IL-6**(pg/mL)			
At baseline	51±2.3	48±2.1	ND
After intervention	68±2.6	50±3.1 b	ND
P values	p < 0.01	p < 0.05	----
**IL-10**(pg/mL)			
At baseline	43±1.8	38±1.7	20±0.7 b
After intervention	70±4.8	35±1.8 a	21±1.1 a
P values	p < 0.001	p < 0.05	P<0.05

The CRP serum levels showed significant differences between PI and HG and also between PI and PC after intervention (p < 0.001). Changes in CRP levels between initiation phase and the final phase were significant in PI group (p < 0.01). After the intervention, serum levels of IL-1*β *declined significantly in PI (p < 0.01) and a significant difference between PI and PC was observed (p < 0.01). After 8 weeks of yogurt consumption, serum levels of TNF-α were declined to 225.48±4.7 pg/mL in PI, that was statistically significant (p < 0.001). 

In addition, serum levels of TNF-*α *in comparison to each other had significant difference after intervention (p < 0.001). After study cessation, serum levels of IL-6 were significantly different between PI and PC (p < 0.01). IL-6 elevation within PI was statistically significant (p < 0.01) and the alterations in PC group were not significant before and after the intervention (p > 0.05). After 8 weeks of intervention, serum levels of IL-10 reached to 70.60±4.82 pg/mL in PI that was a significant enhancement (p < 0.001), but it didn’t change in two other groups (p > 0.05). Significant differences were seen in IL-10 serum levels between PI and HG, and also between PI and PC at the end of the study (p < 0.001). 

## Discussion

Our data indicate that 8 weeks of probiotic yogurt consumption in IBD patients, led to a significant decline in serum levels of pro-inflammatory cytokines like TNF-*α*, IL-1*β *and also in CRP levels. In addition, an increase was observed in serum levels of anti-inflammatory cytokine IL-10 and also in serum levels of IL-6. 

The CRP serum levels were significantly higher in IBD patients in comparison to healthy people at the baseline. At the end of this study, the CRP serum levels were declined significantly in PI group, but despite this decline, it was still higher than healthy control group. A previous study reported that CRP increased in all of CD patients and in 50% of UC patients (22). CRP is a fast and safe method in terms of histological or endoscopical approaches for diagnosis of CD. However, the detection of CRP levels could be a subclinical criterion just for confirmation of disease and not as a predictive indicator for upcoming occurrences (23, 24). Evidence suggested that there was a correlation between CRP and index of disease activity (23, 25). So measurement of CRP in IBD patients for evaluation of the efficacy of treatment on inflammation is a good criteria, such that its decline showed positive effects of therapies and increased values are due to failure in immuno-regulation (24). As our results indicated a decline in CRP levels in IBD patients who consumed probiotic yogurt, but not in other two study groups, this decline could be considered as a favorable outcome. 

The IL-1*β *and TNF- *α *are two key pro-inflammatory CKs (26, 27).The serum levels of IL-1*β *and TNF-*α *in healthy controls in our study were below the sensitivity levels of our assay, but other studies have reported an increase in their levels in IBD patients in comparison with healthy people especially for TNF-*α *levels (26, 28). TNF-*α *level in IBD patients showed 390 folds higher levels compared to healthy group. In this study there was a significant difference also in TNF-*α *concentration between active and inactive IBD courses.This difference was stronger for UC patients. TNF-*α *concentration in active UC patients was 1.7 fold higher than inactive UC (28).

In PI group both these CKs serum levels decreased significantly after intervention. Other studies examined the effect of probiotic on these CKs too. It has been found in the study that in probiotic group, the levels TNF-*α*, IL-1*β *and INF-*γ *cytokines were decreased compared to healthy controls (29). Another clinical trial has investigated the effects of one month probiotic yogurt consumption in 20 IBD patients versus 20 healthy people, in inflammation status. The probiotic yogurt contained *Lactobacillus Rhamnosus GR-1 *and also *L.reuteri RC-14 *in its content. The results of the study represented that the percentage of immune cells like monocytes that produced TNF-*α *declined in both study groups. But contrary to our results, the TNF-α level didn’t show any significant change in IBD group, but the serum levels of TNF-α decreased in control group (30).

An RCT study in UC patients that evaluated a symbiotic containing *Lactobacillus Paracasei B 20160 *showed an increase in serum levels of TNF-*α*, but no difference in IL-1β values at baseline. Interestingly, after intervention no changes in TNF-α serum and mRNA levels were observed. The expression of TNF-*α *and IL-1*β *in lymphocytes slightly decreased but it was not significant too (31). Another RCT study upon 30 UC patients showed that the mRNA expression of some cytokines was changed during the study after the intervention in particular the expression of inflammatory cytokines TNF-*α *and IL-1*β *declined (32). The oral consumption of *Lactobacillus suntoryeus HY7801 *in TNBS-induced colitic mice has caused a significant decline in expression of IL-1*β*, TNF-*α *and IL-6 in colon (33). Biopsies obtained from the colon of patients with active UC were cultured with *Bifidobacterium *and a lower concentrations of TNF-*α *in epithelium cells cultured with probiotic. In addition they observed lower numbers of NF-κB positive cells in cells cultured with probiotics compared with cells cultured alone (34). Another study of oral probiotics intervention in UC patients showed a significant decrement in the expression of NF-κB and TNF-*α *mRNA after intervention (35).

NF-κB is a transcription factor which plays a key role in inflammatory conditions and specifically secretion of inflammatory cytokines. Generally, NF-κB can be found in the cell cytoplasm, in inactivated form, sticking with IκB, which is an inhibitory protein. When an inflammatory state occurs in body, IκB will be phosphorylated with related kinases and then protein dispart and breakup happens. In the next steps NF-κB shifts to the nucleus and starts up its activities by joining to specific areas of DNA on specific genes (34). There is a multidimensional relationship between NF-κB and pro-inflammatory cytokines. For example when the level of pro-inflammatory cytokine TNF-*α *or IL-1 elevates, it can heighten the NF-κB action, and then NF-κB affects positively on the expression of cytokines like TNF-*α *itself and also IL-6, IL-8 and others; so the inflammatory process exacerbates (34). Beside these effects of NF-κB, it has been shown that NF-κB can prevent the apoptosis of certain immune cells like macrophages and neutrophils, so it can amplify the damages caused by inflammation particularly in intestinal epithelium (35). 

There are evidences about the role of NF-κB in pathways that many probiotics effect on cytokines which take place via them with modulating this transcriptional factor (20). Interferential effects of some probiotics mediated through IκB/NF-κB inflammatory pathway in epithelium of intestine, and changed the degradation of IκB (36). The results of an RCT with probiotic cachet supplementation in UC patients showed an apparent IκB breakup inhibition in cytoplasm of the cells in the intervention group. Along with this alteration, there was seen a significant decline in NF-κB expression and activity in the nucleus of colonic cells (32). 

There are evidences about the elevation of IL-6 in IBD in different disease course. According to a study, IL-6 concentrations were higher in both relapse and remission courses in comparison with healthy group (37). Another study showed elevated IL-6 levels in active phase of IBD that returned to normal level in remission phase (38). 

One of the approaches to eliminate the inflammation in inflammatory diseases, is theraupeutic agents against proinflammatory cytokines including IL-6. However recent evidence suggests that IL-6 could have regenerative or anti-inflammatory properties as well as its well-known proinflammatory ones (39). According to some studies, the binary role of IL-6 on inflammation could be due to two different signaling pathways (classic and trans-signaling) on target cells which is determined by type of target cells. Classic signaling pathway occurs in cells that could express gp130 (a glycosylated type I membrane protein) and the IL-6R (IL-6 binding type I transmembrane glycoprotein). It was found in animal models that classic transduction is an anti-inflammatory pathway and it could be mediated by a membrane bound IL-6 receptor (mbIL-6R) that was expressed in intestinal epithelial cells. In chronic inflammatory diseases like CD, IL-6 acts via trans-signaling pathway and leads to aggravation of monocytes (39, 40). It is not reported which pathway is involved in probiotic effects. But according to our results it’s possible that the probiotics could affect classic IL-6 pathway, but it needs to be further investigated.

Researchers showed that attenuation of proinflammatory cytokines like TNF-*α *and inflammation in probiotic fed IL-10 KO animal models caused by mechanisms that were independent of anti-inflammatory cytokine IL-10 (41). In the present study the TNF-*α *levels were deceased after the intervention in PI group. Whilst such decrease was accompanied by a significant change in IL-10 levels at this point, it was not obvious if these changes were related to each other or not.

In IBD, IL-10 is up-regulated because of its anti-inflammatory effect. IL-10 serum concentrations decrease inflammation in mucosa but it is not strong enough to suppress it (42). In the present study the serum levels of IL-10 were significantly higher in IBD patients than healthy control group. This significant relation remained after the intervention, but in a stronger degree. 

It has been demonstrated that one of the ways that probiotics represent their immunomodulatory effects is through the increase of IL-10 levels, but this effect isn’t true about all strains and doses and also there is no certainty about the effective range of IL-10 level as an anti-inflammatory agent (20, 38). One of the speculations in this regard is the Probiotic inducing effect on dendritic cells. These cells could enhance anti-inflammatory cytokines secretion such as IL-10 (43).

Another proposed factor in this regard is Toll-like receptors (TLRs). TLRs are trans-membrane receptors involved in immunomodulation. When a threatening situation occurs, the increase in TRL signaling evoke important immunological responses and also enhance the NF-κB signaling, but prolonged TLR activation may lead to inflammation and further damage itself (44, 45). TLRs are involved in the activation of innate immune system in the intestinal epithelium. On the other hand, it has been mentioned that the probiotics exert their avails predominantly on innate immune system, so it has been suggested that maybe one of the mechanisms of probiotics is making alterations in TRL expression (20). There are few data in this regard specially from clinical studies, but an example of animal researches is the study in TNBS-induced colitic mice administrated Lactobacillus HY7801 constructed an inhibition in the activity of NF-κB and TLR-4 expression (33). But it is obvious that the clarifying role of TLRs in this context needs more investigations. There have been limitations in our study; first of all, our patients weren’t followed after intervention, so it wasn’t possible to evaluate the long term effects especially on remission duration. Secondly, the heterogeneity of disease type, duration and medications, was one of our limitations to generalize our results to all IBD patients. Although when our patients were all in remission phase, they usually consumed similar categorical drugs. Finally we couldn’t record the patients’ daily symptoms alterations during intervention to detect suitable clinical outcome of our intervention.

Despite of encouraging results in the present study, prescription of probiotics as a medical intervention needs more well-designed trials. However, it can be claimed that intestinal homeostasis is a balance between pro and anti-inflammatory responses of intestinal immunocytes and possibly could be maintained by probiotics.
